# Appendiceal Diverticulitis in a Young Female Diagnosed on Pathology after Laparoscopic Appendectomy for Acute Appendicitis

**DOI:** 10.1155/2021/2508956

**Published:** 2021-03-08

**Authors:** Oluwatobi O. Onafowokan, Aboubakr Khairat, Hugo J. R. Bonatti

**Affiliations:** ^1^University of Maryland Community Medical Group, Easton, MD, USA; ^2^Royal Lancaster Infirmary, Lancaster, UK; ^3^Meritus Surgical Specialists, Hagerstown, MD, USA

## Abstract

*Background*. Appendiceal diverticulitis is a rare cause of inflammation of the appendix, which may mimic acute appendicitis. Its diagnosis is often delayed, and its occurrence carries an increased risk of significant complications, such as perforation. *Case Presentation*. A 23-year-old woman presented with sudden onset, severe, right lower quadrant abdominal pain and nausea. Her WBC was elevated, and abdominal CT showed findings indicative of acute appendicitis with a 13 mm fluid-filled appendix and local stranding. During laparoscopic appendectomy, significant inflammation was found around the appendix with some mucous material around the tip. The appendix base was not involved, and an endoloop was used to secure the stump. No other intra-abdominal abnormalities were observed. The patient recovered uneventfully. Pathology showed no classic appendicitis but appendiceal diverticulitis with signs of perforation. *Discussion*. Appendiceal diverticulitis is a rare condition which cannot be distinguished from acute appendicits clinically and on imaging. Diagnosis may be established based on pathology such as in our case. Appendectomy is indicated in appendiceal diverticulitis, and an appendix diverticulum is incidentally found during surgery or other investigations. This is due to the increased risk of perforation and the reported development of malignant tumors, including the appendix carcinoid.

## 1. Introduction

Appendiceal diverticulosis is an uncommon pathology, first described in 1893 [1–3]. Congenital appendiceal diverticulosis is a true diverticulum, with a rare incidence of 0.014% [4]. Acquired appendiceal diverticulosis is a false diverticulum on the mesenteric border of the appendix, with a relatively more common incidence of 1.9%. Pathogenesis of acquired appendiceal diverticulosis is not completely understood, but it may be associated with diverticulosis of other colonic segments, and various pathologies of the appendix [3, 5].

Appendiceal diverticulitis occurs due to the inflammation of an appendiceal diverticulum [2, 6]. It is a rare cause of inflammation of the appendix, which may mimic acute appendicitis [5, 7]. Its diagnosis may be delayed, and its occurrence carries an increased risk of significant complications, including perforation [8] and a higher risk of mortality [2, 4, 9]. Progression of diverticulosis to diverticulitis may occur following a partial or complete obstruction of the appendix lumen. This obstruction may be due to inflammation, mucosal swelling, fecaliths, torsion, or fibrous strictures [2].

Appendectomy is recommended when appendiceal diverticulosis is incidentally discovered on imaging or during surgery as up to 66% of patients will progress to an acute inflammation [2]. Other authors have considered prophylactic appendectomy less beneficial [10]. The predominant scenario in the majority of publications involves patients diagnosed with acute appendicitis and undergoing appendectomy, but with final pathology showing appendiceal diverticulitis and not appendicitis. It should be noted that both inflammatory processes may occur simultaneously according to the classification proposed by Lipton et al. [8].

We report the clinical course of a young female who presented with an episode of right lower quadrant pain with a clinical and computed tomography (CT) scan diagnosis of acute appendicitis. She underwent laparoscopic appendectomy and was found to have appendiceal diverticulitis on final pathology.

## 2. Case Presentation

**Figure 1:**
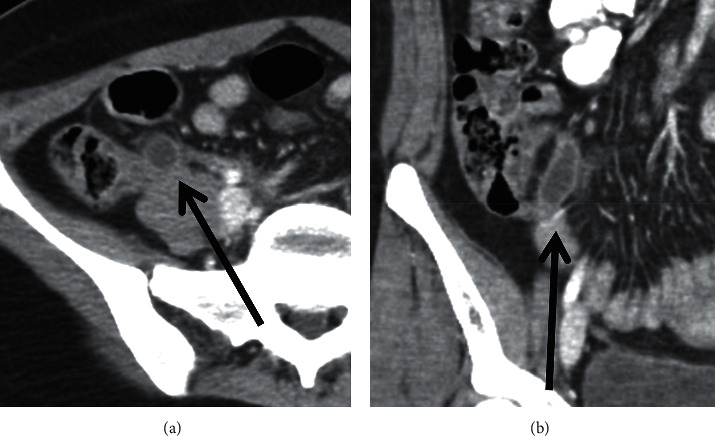
CT scan (transverse and coronal cut). Thickened fluid-filled appendix with localized fat stranding; an appendiceal diverticulum cannot be seen.

A 23-year-old healthy woman presented to the emergency room (ER) with acute onset, severe right lower quadrant (RLQ) pain and nausea. On examination, she was alert and oriented, her abdomen was soft, but she had significant tenderness in the RLQ and a positive Murphy's sign. White blood count was normal, and abdominal computed tomography (CT) scan showed a fluid-filled and dilated appendix with thickened walls and localized inflammatory changes in the RLQ indicative of acute appendicitis (Figure 1).

**Figure 2:**
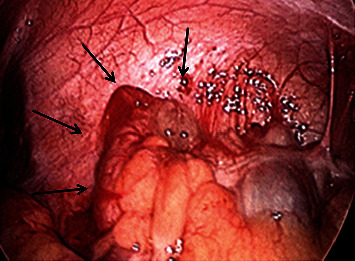
Intraoperative findings: periappendicitis and acute inflammation of the appendix with mucinous secretions.

She was started on antibiotics in the ER (ertapenem 1 g) and consented for laparoscopic appendectomy. Laparoscopy was done with 5 mm left (L) UQ and umbilical trocars and a suprapubic Teleflex MiniLap Alligator Grasper [11]. The appendix was dilated and acutely inflamed with mucosal secretions on the tip of the appendix (Figure 2). The vascular pedicle and appendix base were secured with endoloops.

**Figure 3:**
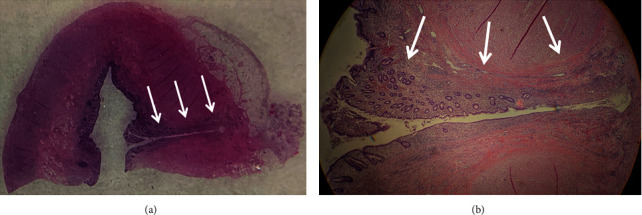
Pathology findings: section of the appendix tip (a) and close up (b) of the diverticulum (arrows) showing acute inflammation.

The patient was discharged the next morning and had an uneventful recovery. Final pathology revealed a diverticulum in the tip of the appendix, with active inflammation (Figure 3).

## 3. Discussion

Acute appendicitis due to appendiceal diverticulitis is a rare condition, with an incidence of 0.004–2.1% [7]. Risk factors may include age >30 years old, male gender, and comorbidities such as cystic fibrosis [7]. Diagnosis is most frequently incidental but may be made my ultrasound or CT scan in selected cases [9]. Appendiceal diverticulitis has been shown to be more than four times as likely to perforate compared with acute appendicitis, increasing mortality 30-fold compared with simple appendicitis [2, 8]. Therefore, correct diagnosis and urgent management with antibiotics and appendectomy are currently favoured with laparoscopy being the preferred approach [12, 13]. In contrast to diverticulitis of the colon or small bowel, no large series demonstrating successful nonoperative management of appendiceal diverticulitis are available, and this is most likely due to the fact that appendicitis and appendiceal diverticulitis are difficult to distinguish on imaging [14]. Radiographic diagnosis of appendiceal diverticulosis/diverticulitis is difficult, but CT scan may identify an appendiceal diverticulum with the pericaecal fat showing increased density.

It should be recognized that diverticula of the appendix are also associated with an increased risk of appendiceal neoplasms including carcinoid tumors, adenocarcinoma, and mucinous adenomas (pseudomyxoma peritonei) [4]; strengthening the argument for appendectomy [13, 15].

To summarise, acute appendicitis due to appendiceal diverticulitis is an uncommon condition, which is often diagnosed on pathology. Appendiceal diverticulitis may be associated with significant complications. Currently, appendectomy is favoured for acute appendiceal diverticulitis and incidentally discovered appendiceal diverticulosis, with laparoscopy being the preferred approach.
